# A review of osteoarthritis signaling intervention using small-molecule inhibitors

**DOI:** 10.1097/MD.0000000000029501

**Published:** 2022-08-12

**Authors:** Junyong Park, Sang Yeob Lee

**Affiliations:** a Division of Rheumatology, Department of Internal Medicine, College of Medicine, Dong-A University, Busan, Republic of Korea.

**Keywords:** clinical trials, investigational treatment, osteoarthritis

## Abstract

Numerous small-molecule inhibitors (SMIs) have been approved as adjuvant or first-line therapies for malignancies. Based on cancer treatment using SMIs, next-generation SMIs that can be used to optimize the therapeutic index, overcome drug resistance, and establish combination therapies are in development. Osteoarthritis (OA) is the most common chronic joint disease with senescence, and there are various approaches to OA treatment; however, the gold standard treatment is controversial. Therefore, in this manuscript, we demonstrated the potential of using SMIs in OA treatment and described the general strategies for using SMIs in OA treatment.

## 1. Introduction

A small molecule with a low molecular weight (<900 Da) is measured on the order of 1 nm and regulates biological functions. It can be applied to regulate cell-signaling molecules, medical drugs, and pesticides for farming. Small molecules are pharmacologically restricted to enzymes that bind specific biological micromolecules and alter the activity or function of the target^[1]^; small molecules are also used as research tools to probe biological functions and new therapeutic agents, and certain molecules can activate a protein with a specific function or disrupt protein-protein interactions. The focus of small-molecule inhibitor (SMI) development has shifted toward the identification and targeting of molecular drivers of cancer. SMIs vary in selectivity, and by virtue of their small size, can potentially bind a wider range of extracellular and intracellular targets in cancer. The US FDA has approved 62 SMIs and nearly these SMIs are effective when administered orally, excerption for netarsudil (which is given as an eye drop), and temsirolimus (which is administered intravenously).^[2]^ Most approved SMIs target intracellular kinases that regulate cell signaling through the transfer of phosphate groups to target proteins.^[3]^ In most cases, SMIs are currently used for cancer treatment. However, their use in noncancer diseases has emerged as a promising alternative treatment. Various neurological, endocrine, inflammatory, and autoimmune diseases are currently being studied for prospective small-molecule targets. There is also a growing body of research suggesting the use of SMI in various autoimmune and inflammatory diseases because of their shared pathophysiology with immune-mediated cellular processes. In the treatment of inflammatory diseases, there are several types of protein targets that could be modulated by SMIs: the regulatory target of inflammatory mediator production, the signaling molecules, and the receptors in the nucleic acid sensing process, and cytokines on the cell surface. Therefore, targeting SMI may be a sequential target therapy for osteoarthritis (OA) treatment.

## 2. Overview of Osteoarthritis

**Figure 1.**
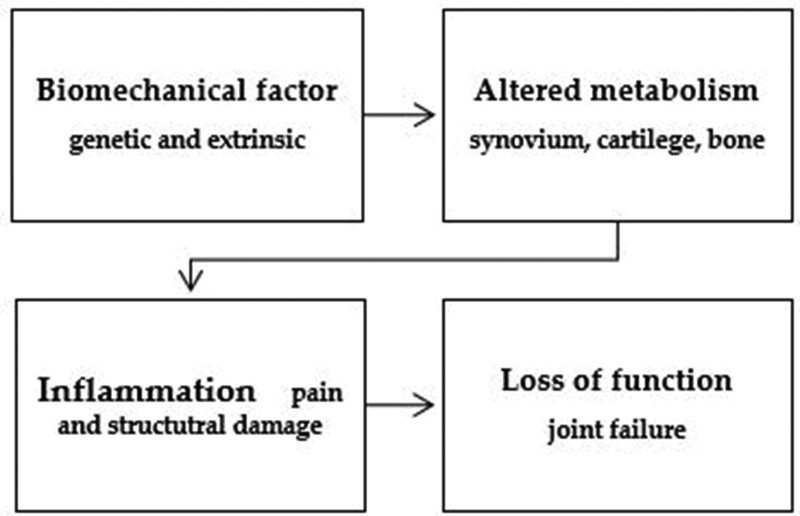
Multiple factors that predispose to, initiate, and perpetuate osteoarthritis.

Osteoarthritis (OA), the most common chronic joint disease, increases with age. The majority of individuals with OA are older than 65 years of age. Cell senescence in joint tissues may contribute to OA development, but multiple factors are involved in OA development (Fig. 1). For example, an increase in inflammatory cells leads to enhanced production of inflammatory cytokines and matrix metalloproteinases (MMPs) in the OA joint.^[4,5]^ Moreover, reduced growth factor levels and chondrocyte degeneration inhibit the repair of matrix synthesis machinery.^[6]^ In addition to the aging process, environmental, biomechanical, and biochemical factors can also contribute to the initiation of OA.^[7]^ OA is associated with all joint structures, such as the meniscus, articular cartilage, synovial membrane, subchondral bone, and infrapatellar fat pad (IFP). The common structural characteristics of OA are cartilage degradation, subchondral bone cysts, osteophytes, and degeneration in the joint capsule and synovium.^[8]^ OA patients have severe joint pain and stiffness, and significantly reduced mobility, leading to decreased quality of life and increased socioeconomic burden on the family and community of patients with OA.^[8]^ The prevalence of OA increases with age; therefore, the worldwide aging population makes OA a non-negligible issue.^[8]^ The treatments currently available for OA are symptom-relieving therapy and surgery for severe OA cases. Therefore, drugs that treat the underlying pathogenesis of OA are not currently available.

## 3. Pathophysiology of OA

### 3.1. Involvement of inflammatory cytokines and chemokines

Chondrocytes and synoviocytes in the joints of patients with OA produce inflammatory cytokines and chemokines. Moreover, synoviocytes produce proinflammatory cytokines and matrix-degrading enzymes in OA,^[9]^ and IFP contains a significant number of immune cells, such as macrophages and T cells. Therefore, IFP plays a role as an inflammatory mediator in OA knees.^[10]^ These changes induce activation of inflammatory cascades. Thus, more inflammatory cytokines and enzymes are released. As a result, the anatomical and physiological environments of the affected joint changes.^[11]^ In OA pathogenesis, IL-6 and IL-1β, secreted from neutrophils and macrophages, are the most common representative cytokines.^[12]^ Increased migration of immune cells (macrophages, B lymphocytes, T lymphocytes, and neutrophils) in the OA synovium, especially within the subintimal layer, is a characteristic of OA.^[13]^ Immune cells and inflammatory mediators secreted from IFP also modulate other OA joint tissues, and these mediators can amplify the OA inflammatory process.^[14]^ IL- 1β and IL-6, secreted from neutrophils and macrophages, can stimulate the increased production of proinflammatory cytokines in IFP.^[15]^ Proinflammatory cytokines secreted by immune cells play a major role in inflammatory conditions. IL-1β and TNF-α are important proinflammatory cytokines in early OA, and these cytokines can drive the inflammatory cascade independently or collaborate with other cytokines in OA pathogenesis.^[16,17]^ These proinflammatory cytokines are produced by activated chondrocytes, synoviocytes, and mononuclear cells.^[18]^ TNF-α and IL-1β have also been used to stimulate inflammation in chondrocyte and synoviocyte cultures. Upon stimulation, activated chondrocytes and synoviocytes release IL-1β, IL-6, IL-8, IL-10, and TNF-α.^[19,20]^ An OA patient study showed that the levels of IL-1β, IL-6, IL-8, IL-18, IL-17, IL-22, and transforming growth factor-beta 1 (TGF-β1) were elevated in the inflamed synovial tissues compared to normal control tissues.^[11]^ Chemokines constitute a subfamily of cytokines with low molecular weight, and chemokines are classified into 4 families, namely, the CXC, CC, C, and CX3C families, according to the position of cysteine (C) residues.^[13,16]^ Chemokines function as chemoattractants and induce the direct migration of immune cells to damaged or infected sites.^[9]^ Increased C-C motif ligand 5 (CCL5), CCL20, CC motif receptor 5 (CCR5), and CCR7 participate in the recruitment of various types of T helper cells such as T helper cell type 1 (Th1), T helper cell type 17 (Th17), and T helper cell type 22 (Th22), which are more highly expressed in affected OA joints.^[11]^ Furthermore, proinflammatory cytokines such as IL-1β, IL-17, and IL-22 are released from immune cells and trigger the inflammatory process in the affected joint. Several studies have shown high CCL2 levels in patients with OA.^[12,21]^ CCL2 is known as monocyte chemoattractant protein-1 (MCP-1). CCL2/CCR2 signaling triggers the recruitment of monocytes in inflamed OA joints and cause inflammatory propagation.^[21]^ Increased CCR2 levels and decreased CXCR3 expression were observed in the peripheral blood of OA patients compared to those of normal control group participants.^[16]^ Increased level of CCR3 and its ligand, CCL11, which is also known as eotaxin-1, have been detected in synoviocytes of OA patients. CCR3 is expressed in T cells and dendritic cells.^[11,12]^ CCL11 stimulates MMP-9 secretion in OA patient synoviocytes. The meniscus and synovial membrane in OA patients showed elevated CCL21 and CCL5 levels in cocultures of an in vivo study.^[22,23]^ CCL2 is also secreted by damaged chondrocytes and synoviocytes. It also recruits CCR2-expressing monocytes to the injured OA joints. CCL7 is a monocyte chemoattractant that has been shown to be increased in the synovial fluid of OA patients,^[13]^ and CCL7 levels in synoviocytes are enhanced by IL-17 expression.^[24]^ CCL20 and its receptor, CCR6, are highly expressed in OA cartilage compared to normal controls, and these chemoattractants stimulate IL-6, MMP-1, and MMP-13 secretion.^[4]^ Moreover, conditioned media obtained from OA tissues, such as cartilage, IFP, meniscus, and synovium, enhanced the production of CXCL8 and CCL21 in vitro.^[9]^

### 3.2. Involvement of matrix metalloproteinases

MMPs constitute a family of zinc-dependent enzymes that regulate the degradation of the extracellular matrix (ECM) via cleavage of peptide bonds in target proteins.^[25]^ MMPs can be classified into several groups based on their structure and substrates: collagenases (MMP-1 and MMP-13), gelatinases (MMP-2 and MMP-9), stromelysins (MMP-3), metalloelastase (MMP-12), matrilysin (MMP-7), and membrane-type matrix metalloproteinases (MT-MMPs).^[26]^ These substances can degrade the ECM of OA cartilage, which is mainly composed of collagens and proteoglycans. MMP-13, MMP-3, MMP-2, and MMP-9 are increased in an OA model cell line.^[20,22]^ Furthermore, MMP-13, a collagenase, plays a pivotal role in the degradation of type II collagen, which is the most abundant collagen type in articular cartilage.^[25]^ The expression of MMP-13 is increased by stimulated expression of CCL20 and interferon regulatory factor-8 (IRF-8) in the chondrocytes of OA patients.^[27]^ MMP-1 is a collagenase that can be elevated in OA.^[11]^ MMP-2 and MMP-9 can cleave ECM proteins, which activate cytokines and chemokines.^[25]^ Moreover, the levels of MMP-2 and MMP-9 are increased to a greater extent in OA synovial tissues than in control tissues.^[28,29]^ MMP-3, known as stromelysin-1, cleaves type II collagen and aggrecan. The chemokine CX3CL1 induces MMP-3 production in OA synoviocytes, and several reports have shown increased MMP-3 levels in OA joints.^[25]^ MMP-10, known as stromelysin-2, has also been shown to be increased in early- and end-stage OA joints.^[30]^ In particular, MMP-13 plays a pivotal role in degrading a disintegrin and metalloproteinase with thrombospondin motifs (ADAMTS)-4 and -5, and degrading aggrecan. Aggrecan and collagen are the major structural components of cartilage ECM, and their degradation is correlated with OA progression. Several ADAMTSs, in addition to ADAMTS-4 and -5 are also expressed in the ECM, and their roles in OA pathophysiology are emerging.^[31]^

### 3.3. Involvement of nuclear factor-kappa B

The nuclear factor-kappa B (NF-κB) transcription factor plays a significant role in inflammation in OA pathogenesis.^[32]^ NF-κB is triggered by proinflammatory cytokines and ECM degradation products.^[32]^ Activated NF-κB modulates the expression of various cytokines, chemokines, and matrix-degrading enzymes, and this function of NF-κB suggests its role in regulating the inflammatory mechanism of OA.^[9]^ The NF-κB signaling pathway is initiated by the activation of IκB kinase (IKK), resulting in phosphorylation and degradation of IκBα by the proteasome. Subsequently, the p65 protein is released, phosphorylated, and subsequently translocated from the cytoplasm to the nucleus. These sequential events promote the expression of MMP-13 and IL-6.^[32]^ Therefore, phosphorylated p65 (p-p65) levels were increased, whereas IκBα levels were decreased in OA.^[10,22,27]^

### 3.4. Involvement of multiple signaling pathway

Enhanced p-p38, p-JNK, and p-ERK in OA provide evidence for the involvement of the mitogen-activated protein kinase (MAPK) signaling pathway.^[33]^ Furthermore, MAPK regulates downstream proinflammatory cytokines and MMPs. MAPKs act as pain mediators and upstream activators. The pathway also involves intracellular MAP kinases (MKKs), which phosphorylate specific MAP kinases.^[33]^ Activated MAPK can activate other protein kinases and transcriptional regulatory proteins, resulting in the upregulation of several inflammatory enzymes and cytokines, such as MMPs, IL-1, and TNF-α. These cytokines can maintain JNK activation and cause greater cytokine and MMP production.^[32]^ PI3K/AKT signaling is activated by IL-1β when it binds to its receptor on the cell surface, which may have a synergistic effect on the NF-κB signaling pathway. Activation of the PI3K/AKT pathway enhances MMP production in OA chondrocytes.^[33,34]^

### 3.5. Involvement of nitric oxide

The nitric oxide (NO, inducible nitric oxide synthase [iNOS]) and prostacyclin pathways (cyclooxygenase-2 [COX-2] and prostaglandin E2 [PGE2]) are also important in OA pathogenesis. IL-1β can induce the upregulation of iNOS and COX-2 in OA pathogenesis, leading to increased production of NO and PGE2.^[35]^ Elevated NO levels inhibit the synthesis of collagen type II (Col2) and proteoglycan.^[36]^ Activated PGE2 inhibits OA chondrocyte proliferation and reduces ECM synthesis.^[37]^ In addition, IL-1β can stimulate A disintegrin and metalloproteinase with thrombospondin motif (ADAMTS-5) production, which can provoke aggrecan degradation.^[37]^ Levels of iNOS, NO, COX-2, PGE2, ADAMTS-5, ADAMTS-4, and VEGF were reported to be increased in an OA animal model, leading to elevated production of inflammatory factors and ECM degradation.^[37,38]^ The expression of runt-related transcription factor 2 (Runx2), an osteogenic transcriptional activator, was found in an OA pathogenesis study.^[39]^ Bromodomain-containing protein 4 (BRD4) is involved in the NF-κB signaling pathway.^[17]^ Therefore, inhibition of BRD4 suppresses IL-1β-induced expression of proinflammatory cytokines and phosphorylation of p65. TGF-β1-induced NGF expression is dependent on the activin receptor-like kinase 5-Smad2/3 (ALK5-Smad2/3) signaling pathway. ALK5 is a transmembrane receptor of TGF-β, and activated ALK5 triggers downstream signaling cascades via the Smad-dependent pathway.^[4,32,40]^

### 3.6. Involvement of micro RNAs

MicroRNAs (miRNAs) are short, endogenous noncoding RNAs that regulate posttranscriptional gene expression by attaching to the 3′untranslated region (3′UTR).^[41]^ Once miRNAs bind to target genes, they can inhibit the translation process or attenuate the stability of messenger RNAs (mRNAs) of a wide array of genes, including those involved in OA pathogenesis.^[41,42]^ miR-146a and miR-126 are upregulated in OA chondrocytes and are activated by lipopolysaccharide (LPS) and IL-1β, respectively.^[35,43]^ miR-146a targets CXCR4 and downregulates CXCR4 expression. miR-381a-3p and miR-454 are upregulated in OA pathogenesis.^[43,44]^ Moreover, miR-149 may be bound to the transforming growth factor-1-activating kinase 1 (TAK1) 3′UTR.^[45]^ TAK1 expression increased with decreased miR-149 expression in OA. The NF-κB signaling pathway is downstream of TAK1. Therefore, activated TAK1 can increase NF-κB activation in OA pathogenesis.^[46]^ P2X7R is expressed in OA, and P2X7R induces OA chondrocyte proliferation and the expression of IL-6 and IL-8. Furthermore, P2X7R is an adenosine triphosphate (ATP)- gated plasma membrane ion channel. Damaged immune cells produce ATP, leading to activation of P2X7R. Therefore, P2X7R plays a role in IL-1β maturation and secretion from activated immune cells.^[47]^ miR92a-3p directly targets the 3′UTR of ADAMTS-4 and ADAMTS-5 mRNAs,^[37]^ and miR-502-5p could also target the 3′UTR of TNF receptor-associated factor 2 to inhibit its expression.^[48]^

## 4. Current OA treatment and the need for novel OA drugs

The current approaches for OA treatment are largely palliative. To relieve the natural course of OA, modifying OA bone structural damage and controlling pain in tandem have become the focus of OA treatment development. The management of OA treatment involves pharmacological and nonpharmacological strategies, and joint replacement surgery is considered for symptom-refractory OA. Pain control remains the mainstay of pharmacological treatment for OA, including paracetamol, topical and oral nonsteroidal anti-inflammatory drugs (NSAIDs), and opioid medications. However, the benefits of paracetamol and opioids are limited, and NSAIDs are not suitable for many patients because of their side effects. Intra-articular therapies, such as corticosteroids or hyaluronic acid applications, are also commonly used, although often with short-term benefits. OA drugs that modify OA bone structural damage and block OA progression are referred to as disease-modifying OA drugs (DMOADs) and play a role in delaying OA progression, completely stopping the natural course of OA, or regenerating damaged OA joints. In the past, glucosamine sulfate was used as a DMOAD for OA management. However, the literature to date indicates that it has not been proven to slow OA progression or effectively decrease OA-associated pain. Therefore, no DMOADs are currently licensed for OA treatment, although several potential treatment drugs are under investigation.^[7]^ Moreover, OA therapeutic development is limited by several factors, such as bioethics, the naturally slow course of OA, disease heterogeneity, the poor association between structural damage and clinical symptoms, and inconsistency between human models and animal models. However, much research is largely focused on more targeted therapies than palliative drugs and can be locally administered, specifically via intra-articular injections, which maximize local efficacy while reducing systemic toxicity. Intra-articular capsaicin is a protein expressed on nociceptive nerve fibers (Aδ and C), and its activation leads to a prolonged refractory state known as desensitization. Therefore, it is an attractive target for local analgesic treatments. Recent trials targeting the blocking of peripheral nociceptive pain pathways in OA have demonstrated promising analgesic effects in knee OA. An antinerve growth factor monoclonal antibody, Tanezumab, targets nerve growth factor, thereby preventing nerve growth factor from binding its receptor, with the overall aim of reducing OA knee pain.^[7]^

## 5. Clinical trials of small-molecule inhibitors in OA treatment

Drugs for treating OA can be classified into drugs that improve joint pain or symptoms and improve joint structure, or slow disease progression, with or without an effect on pain. A variety of potential therapeutics targeting OA inflammation control, cell aging, cartilage degradation, subchondral bone remodeling, and peripheral nociceptive pain pathways are expected for OA treatment over the next few years. Human, double-blind, and randomized controlled trials are expected to verify the safety and efficacy of novel therapies targeting OA.

### 5.1. Therapeutic targeting OA inflammatory mechanisms

Inflammatory cytokines can be detected in OA synovial fluid and OA patient serum, suggesting that inflammation may play a role in OA pathogenesis. Therefore, OA is thought to be a low-grade inflammatory disease. Recently, some reports have shown that low-grade, chronic, sterile inflammation associated with OA is closely related to dysregulation of the immune system with aging. Anti-inflammatory therapeutics and treatment modalities targeting senescence processes may be promising approaches to attenuate the disease progression. IL-1 is expressed in cartilage, synovium, and OA synovial fluid. Thus, drugs targeting IL-1 receptor antagonists, including humanized monoclonal antibodies and human dual variable domain immunoglobulin, have been used in human clinical trials. However, these results obtained to date suggest that IL-1 inhibition is not effective in most patients with OA.^[49]^ Whether subgroups of OA patients will show improved joint pain or benefit from protective effects in human OA joint due to IL-1 inhibition remains an open question. TNF-α is a proinflammatory cytokine produced by OA synoviocytes and enhances the expression of other proinflammatory cytokines, such as IL-6, IL-8, MMP, and NO. Animal studies have suggested that anti-TNF-α therapy has a protective effect on OA cartilage and that inhibitors of TNF-α can be potential candidates for disease-modifying OA drugs.^[50]^ However, several recent randomized, double-blind, placebo-controlled trials for etanercept, infliximab, and adalimumab have not provided evidence to support the use of these OA treatments, but for a therapeutic strategy targeting inflammation, short-term treatment with TNF-α inhibitors during disease flares may be considered.^[51,52]^ Traditional disease-modifying antirheumatic drugs (DMARDs), such as methotrexate and hydroxychloroquine, may have the potential to reduce joint pain and slow structural joint degeneration, considering the increasing recognition that OA represents an inflammatory phenotype. However, some recent randomized double-blind placebo-controlled trials showed that hydroxychloroquine and methotrexate did not relieve OA symptoms or delay OA structural damage.^[50]^ Therefore, more evidence is required to establish the clear role of DMARDs in OA treatment.

### 5.2. Targeting antiaging treatment in OA

Dysregulation of the immune system during aging may play a role in chronic OA inflammation. Age-related mitochondrial dysfunction and oxidative stress may induce senescence in joints with OA. Furthermore, the accumulation of aging cells in OA joints stimulates the secretion of cytokines and chemokines.^[53]^ Therefore, aging cells in OA joints are potential treatment targets for eliminating OA. UBX0101 is a p53/MDM2 interaction inhibitor that can reduce the cytokines and chemokines secreted from OA joint aging cells and might improve overall OA joint function. Several randomized, placebo-controlled clinical trials of UBX0101 have been performed.^[54]^ Curcumin is extracted from *Curcuma longa* root and is known to suppress oxidative stress and inflammation by scavenging active oxygen and inhibiting the NF-κB pathway.^[55]^ A systematic review and meta-analysis of randomized controlled studies showed some beneficial effects of curcumin on OA knee pain and quality of life in patients with knee OA. However, further studies on curcumin are needed to assess its therapeutic effect on OA.

### 5.3. Targeting drugs to cartilage metabolism

In OA pathogenesis, abnormal Wnt signaling leads to increased secretion of catabolic enzymes and inflammation in the OA joint. Animal model studies have demonstrated that Wnt pathway inhibitors can delay OA development; however, excessive inhibition causes OA cartilage degradation and bone destruction. Thus, maintaining homeostasis of the Wnt pathway is a potential therapeutic goal. Notably, a recent phase I clinical trial (NCT02095548) demonstrated the therapeutic potential of SM04690 as a disease-modifying OA drug.^[56]^ Lorecivivint is a small-molecule inhibitor that modulates the Wnt signaling pathway by inhibiting the intranuclear kinases CDC-like kinase 2 and dual-specificity tyrosine phosphorylation-regulated kinase 1 A. Recently, a multicenter, randomized, double-blind, placebo-controlled study showed ameliorated pain, improved joint function, and attenuated OA damage, as shown on radiographs.^[57]^ Cathepsin-K is the predominant cysteine cathepsin in the skeleton and plays an important role in the resorption of cartilage and bone. Several observations have indicated the upregulation of cathepsin-K in OA cartilage and inflamed synovial tissue. The cathepsin-K inhibitors balicatib and MIV-711 have a protective effect on both bone and cartilage structures, and significantly reduce the levels of biomarkers of bone and cartilage degradation (CTX-I and CTX-II).^[58]^ MMPs and ADAMTS have been demonstrated to play critical roles in articular cartilage degradation and are considered potential targets for OA treatment. In preclinical trials, highly selective MMP-13 inhibitors have shown advantages in slowing the progression of OA, and targeting ADAMTS-5/ADAMTS-4 monoclonal antibodies through intra-articular administration was reported to slow OA disease progression in animal models of OA. Although the oral SMI of ADAMTS-4 and ADAMTS-5 are in human clinical trials, the results of these trials have not yet been published.^[59]^ Anticatabolic agents used to delay the progression of cartilage destruction are alternative approaches to cartilage repair for OA treatment. Preclinical data of treatment with sprifermin, which is a recombinant human fibroblast growth factor 18, showed that it bound to and activated fibroblast growth factor receptor 3 in cartilage to promote chondrogenesis, cartilage matrix formation, and cartilage repair. The results from several randomized multicenter clinical trials supported the benefits of intra-articular injection of sprifermin to modify structural progression, and therefore, it may be a potential DMOAD.^[60]^ Overexpression of transforming growth factor-β1 (TGF-β1) has been found to lead to chondrocyte apoptosis and cartilage degeneration with OA-like changes in knee joint tissue in vitro and in OA animal models. Therefore, TGF-β type I receptor inhibitors can inhibit the degradation of articular cartilage. In randomized, double-blind, placebo-controlled phase studies, allogeneic chondrocytes expressing TGF-β1 were delivered by intra-articular injection to an OA knee joint to attenuate pain and improve physical function in OA patients.^[61]^

### 5.4. Targeting drugs to the subchondral bone

Subchondral bone resorption and high bone turnover can play a role in OA pathogenesis. Thus, subchondral bone may be a potential target for OA therapy. However, currently available drugs targeting the subchondral bone have not been approved for the treatment of OA because of inconsistent efficacy or safety. Oral salmon calcitonin, teriparatide, and vitamin D are used in clinical trials as anabolic therapies for subchondral bone in osteoporosis.^[62]^

### 5.5. Targeting drugs for relieve pain relief

New therapies are urgently needed for patients with OA suffering from inadequate pain relief; therefore, monoclonal antibodies that specifically target nerve growth factor and inhibit binding to nociceptive nerve fiber receptors are attractive potential novel analgesic agents. Tanezumab is the most widely studied monoclonal antibody, and the US FDA recently granted certification for its use in the treatment of chronic OA pain and chronic low back pain.^[63]^ However, anti-NGF treatment may aggravate the structural progression of OA in patients with pre-existing subchondral insufficiency fractures.

## 6. Experimental studies for identifying additional small-molecule inhibitors to use in OA treatment

### 6.1. Rationale for employing small- molecule inhibitors for OA treatment

Some SMIs, such as Janus kinase inhibitors, are used for rheumatoid arthritis treatment. SMIs have not been used clinically for the treatment of OA. However, understanding the molecular mechanism underlying the pathogenesis of OA may involve the use of SMIs for treating patients with OA. Various molecules are involved in the regulation of joint homeostasis under healthy conditions as well as in the pathogenesis of OA. Various cytokines, chemokines, and MMPs are secreted from chondrocytes and synoviocytes from patients with OA as well as from healthy persons. In addition, the expression of OA pathogenesis-associated cytokines can be stimulated by the NF-*κ*B, MAPK, PI3K/AKT, prostacyclin, and NO pathways. Hence, these various molecules may be potential therapeutic targets for OA patients, and the recently discovered inflammatory action of miRNAs in the control of joint homeostasis may also provide a therapeutic strategy. Circular RNAs, single-stranded, and noncoding regulatory RNAs may play roles in miRNA sponging and thus inhibit miRNA activity in OA pathogenesis.^[36]^

### 6.2. Suggested potential small-molecule targets in treating OA.

**Figure 2.**
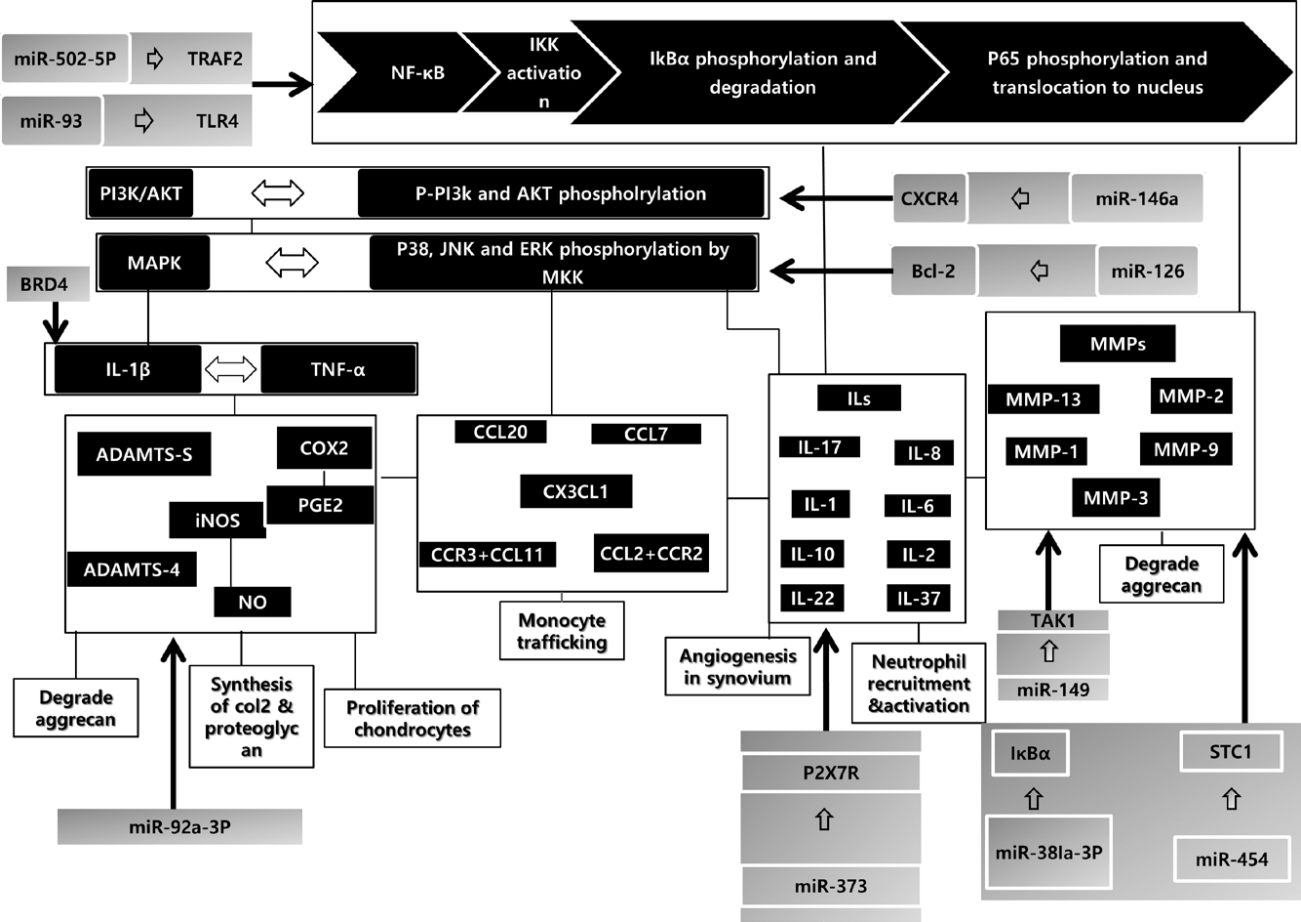
The overview of potential small molecule targeting in osteoarthritis treatment. ADAMTS = a disintegrin and metalloproteinase with thrombospondin motifs, CCL = Chemokine (C-C motif) ligand, COX-2 = Cyclooxygenase-2, IKK = IκB kinase IKK complex, IL = Interleukin, miR = A micro-RNA, MMP = Matrix metalloproteinases, NF-kB = Nuclear Factor kappa-light-chain-enhancer of activated B cells, NO = nitric oxide, P2X7R = P2X purinoceptor 7, PGE2 = Prostaglandin E2, STC1 = Stanniocalcin 1, TAK1 = TGFβ-activated kinase 1, TLR4 = Toll like receptor 4, TRAF2 = TNF receptor-associated factor 2.

To date, the following molecules have been documented as potential small-molecular targets for treating OA (Fig. 2).

#### 6.2.1. IL-6 and signal transducer and activator of transcription-3 (STAT-3).

IL-6 induces chondrocyte catabolism mainly via STAT-3 signaling, a pathway activated in the cartilage of joints affected by OA in the medial meniscus. The systemic blockade of IL-6 by neutralizing antibodies or STAT-3 by a small-molecule Stattic alleviated the need for surgery in medial meniscus-induced OA models.^[64,65]^

#### 6.2.2. TNF-α.

TNF-α, an inflammatory cytokine produced by OA synoviocytes, may play a pivotal role in OA joint structural damage and pain. Furthermore, TNF-α enhanced the activation of several inflammatory cytokines (such as IL-6 and IL-8) and provoked the synthesis of MMP, cyclooxygenase, and NO.^[66]^ Several studies have suggested that anti-TNF-α therapy may play a protective role in OA cartilage by reducing cartilage matrix degradation.^[67]^ Thus, inhibition of TNF-α expression may be considered a potential target for OA treatment.

#### 6.2.3. IL-1α.

A previous study using a computational model of small molecule interactions with the IL-1 family members demonstrated that small molecules are more efficient in inhibiting cartilage degradation by binding directly to IL-1α, but not IL-1α receptors. This study indicates that IL-1α inhibiting small molecules have therapeutic potential for the treatment of OA.^[68]^

#### 6.2.4. G protein-coupled receptor (GPR)-43.

Butyrate reduced the expression of canonical proinflammatory mediators (Nos2, COX-2, and IL-6), proinflammatory adipokines (lipocalin-2 and nesfatin-1), and adhesion molecules (VCAM-1 and ICAM-1) in IL-1β-stimulated chondrocytes, inhibiting several inflammatory signaling pathways (the NF-κB, MAPK, AMPK-α, and PI3K/Akt pathways). Butyrate also reduced metalloproteinase production and limited the loss of type II collagen in IL-1β-inflamed chondrocytes. In IL-1β-stimulated chondrocytes, GPR-43 acts as a target of butyrate activity in chondrocytes. Thus, GPR-43 and other butyrate receptors may be potential small-molecule candidates for treating OA.^[69]^

#### 6.2.5. Nuclear receptor coactivator 3.

The loss of nuclear receptor coactivator 3 (NCOA3)was associated with a greater risk of developing OA. Thus, maintaining NCOA3 function may present a potential therapeutic approach to interfere with OA progression. A previous study showed that the promotion of posttraumatic OA progression was significantly retarded by administration of an NF-κB pathway inhibitor, indicating that enhancement of NF-κB activation, at least partially, induces NCOA3 loss in posttraumatic OA.^[70]^

#### 6.2.6. Transforming growth factor β (TGFβ).

Dysregulated transforming growth factor β (TGFβ) signaling is implicated in OA development, and TGF-β1 strongly induces both pSmad1/5 and pSmad2. TGFβ-activated kinase 1 (TAK1) inhibitors facilitate Smad-dependent signaling. Therefore, SMIs targeting TGFβ-induced pSmad1/5/9-, pSmad2/3-, and TAK1-dependent signaling are therapeutic strategies for OA treatment.^[71]^

#### 6.2.7. Runx family proteins.

Runx family proteins, which are runt-related transcription factors, play crucial roles in OA chondrocyte hypertrophy, and Runx1 has been shown to be an important factor for early chondrogenic differentiation. Runx1 expression is downregulated in both mouse and human OA cartilage compared with normal tissues. The synthetic small compound TD-198946, exerting its effect through the regulation of Runx1 expression, successfully prevented and repaired the degeneration of articular cartilage.^[72]^

#### 6.2.8. P2X purinoceptor 7.

P2X purinoceptor 7 (P2X7R) is a trimeric ATP-activated ion channel gated by extracellular ATP, which plays an important role in numerous disease components of OA, including pain, neurodegeneration, and inflammation. Ligand-based quantitative pharmacophore modeling of P2X7R antagonists and virtual screening and molecular docking procedures have led to the identification of 6 potential molecules that can potentially be used to design novel P2X7R inhibitors.^[73]^

#### 6.2.9. CDC42-actin-myocardin-related transcription factor-A (MRTF-A).

Proteoglycan 4 (PRG4) and tenascin C (TNC) are expressed in superficial zone chondrocytes and have been shown to protect chondrocytes. A study showed that treatment with the CDC42 inhibitor ML141 inhibited the CDC42-actin-myocardin-related transcription factor-A (MRTF-A) signaling pathway. ML141 treatment decreased PRG4 and TNC expression and correlated with increased cell circularity and the G-/F-actin ratio, indicating that the MRTF-A signaling pathway is a potential target for OA treatment.^[74]^

#### 6.2.10. Endothelin-1 (ET-1).

ET-1 induces Oncostatin M (OSM) expression in OA osteoblasts by transactivating the OSM gene promoter. OSM contributes to cartilage degeneration in OA, and is expressed in OA osteoblasts. This outcome is a mechanistic role of ET-1 in the pathophysiology of OA. A study showed that the ETA receptor (ETAR) antagonist BQ123 prevented ET-1 treatment-induced OSM expression, suggesting ET-1 as a treatment tool for OA.^[75]^

#### 6.2.11. Wnt/β-catenin.

Treatment with periostin, an extracellular matrix protein involved in the pathophysiology of OA, increased MMP-13 expression, and promoted cartilage degeneration through collagen and proteoglycan degradation. Periostin induction of MMP-13 expression was blocked by CCT031374 hydrobromide, an inhibitor of the canonical Wnt/β-catenin signaling pathway.^[76]^ Growth differentiation factor 5 (GDF5), a member of the bone morphogenetic protein family, inhibits the expression of OA cartilage extracellular matrix-degrading enzymes. The inhibition of MMP-13 expression through GDF5 stimulation is mediated by the canonical Wnt inhibitor dickkopf 1 (DKK1). Thus, inhibition of MMP-13 expression through GDF5 stimulation may be a treatment strategy for OA.^[77]^ The expression level of the ubiquitin-conjugating enzyme E2 M (UBE2M) was significantly higher in patients with OA in healthy individuals. The apoptosis of human OA chondrocytes was inhibited when UBE2M was silenced and increased when UBE2M was overexpressed. XAV939, a tankyrase 1 inhibitor, blocked the Wnt/β-catenin signaling pathway, which significantly reversed the change introduced by UBE2M. Therefore, the SMI of UBE2M is an option for OA treatment.^[78]^ In addition, an inhibitor of Wnt signaling is a target pathway for regulating OA progression, and Wnt pathway upregulation contributes to knee OA through osteoblast differentiation, increased catabolic enzymes, and inflammation. The small-molecule Wnt pathway inhibitor, lorecivivint (SM04690), modulates the Wnt pathway by inhibiting CDC-like kinase 2 and dual-specificity tyrosine phosphorylation-regulated kinase 1A. Lorecivivint enhances chondrogenesis, chondrocyte function, and anti-inflammatory properties Thus, lorecivivint also shows the potential to modify the structure and improve the symptoms of OA knee.^[57]^

#### 6.2.12. C-X-C motif chemokine ligand 12/C-X-C chemokine receptor type 4 (CXCL12/CXCR4) axis.

A SMI of the CXCL12/CXCR4 axis can attenuate traumatic OA by maintaining transforming growth factor-β1-induced expression of tissue inhibitor of metalloproteinase-3 (TIMP-3) *via* the phosphatidylinositol 3-kinase/Akt pathway. Therefore, inhibition of the CXCL12a/CXCR4 signaling axis may be a treatment strategy for OA.^[79]^

#### 6.2.13. NF-κB.

Ivabradine, a funny current (I_f_) inhibitor, abolished the activation of nuclear factor (NF-κB) by inhibiting the nuclear translocation of NF-κB p65. As a result, ivabradine abrogated TNF-α-induced upregulation of matrix metalloproteinase-3 (MMP-3), MMP-13, ADAMTS-4, and ADAMTS-5 and attenuated TNF-α-induced reduction of both mRNA and protein levels of type II collagen and aggrecan. Ivabradine also inhibited the secretion of IL-6 and IL-1β and the production of reactive oxygen species in chondrocytes.^[80]^

#### 6.2.14. MicroRNAs.

MiR-29b-3p or PGRN was upregulated in cartilage tissue obtained from patients with OA. A previous study showed that miR-29b-3p facilitates chondrocyte apoptosis and OA by targeting PGRN. in vivo, joint cavity injection with a miR-29b-3p inhibitor prior to surgical induction of OA significantly suppressed the upregulation of miR-29b-3p and ameliorated articular chondrocyte apoptosis and cartilage loss in rats with surgically induced OA. MiR-29b-3p or PGRN may be potential targets for OA treatment.^[81]^

#### 6.2.15. Molecular chaperone.

Stress conditions, such as insufficient protein folding in the endoplasmic reticulum (ER), extracellular matrix protein aggregation, ER stress, and the unfolded protein response, are relieved in OA chondrocytes through the initiation of cell death by apoptosis. Persistent ER stress in cartilage has emerged as a pathogenic mechanism of chondrodysplasia and OA. Decreased expression of molecular chaperones during aging induces ER stress and chondrocyte apoptosis in monkey articular cartilage, inducing OA. Treatment of cultured chondrocytes with a small-molecule chemical chaperone, 4-phenylbutyric acid (PBA, a general ER stress inhibitor) or PERK inhibitor I (an ER stress inhibitor specifically targeting the PERK branch of the unfolded protein response pathway), decreased the expression of ER stress and apoptotic markers, and prevented OA. Restoring proteostasis using chemical/molecular chaperones or ER stress inhibitors may be a therapeutic option for treating aged-linked OA.^[82]^ Furthermore, 4-PBA inhibited ER stress development via ERp57. Therefore, 4-PBA may be used alone or in combination with other drugs in therapy for patients with ER stress–related OA.^[83]^ Insulin-like growth factor-1 (IGF-1) promotes matrix synthesis and cell survival in OA cartilage. A high-fat diet reduced IGF-1 mediated proteoglycan synthesis. Treatment of human chondrocytes with palmitate induced the expression of CHOP (a protein marker for ER stress), activated JNK, and inhibited IGF-1 function. 4-PBA alleviates ER stress and rescues IGF-1 function, and a JNK inhibitor rescued IGF-1 signaling.^[84]^

#### 6.2.16. Fibroblast growth factor.

Dysregulated fibroblast growth factor (FGF) signaling, which is mediated through its receptors FGFR1, FGFR2, FGFR3, and FGFR4, causes various human diseases. FGF traps, anti-FGF/FGFR monoclonal antibodies (mAbs), and small-molecule FGFR inhibitors are under development as anti-FGF signaling therapeutics. In addition, FGF analogs are being developed for the treatment of OA.^[85]^

#### 6.2.17. Nerve growth factor.

Nerve growth factor (NGF) is a neurotrophin that activates nociceptive neurons to transmit pain signals from the periphery to the central nervous system and exerts its effects on neurons through tyrosine kinase receptor signaling. The SMIs of NGF receptors can be used for analgesia in chronic OA pain, although the higher tested doses of NGF receptor inhibitors also increased the risk of inducing rapidly progressive OA.^[86]^

## 7. Future direction and conclusion

SMIs have not been used as a part of the current OA treatment strategies. However, they are the basis for rational drug development, and most pharmacotherapies for OA are symptomatic therapies, such as those used for pain control. However, certain SMIs, such as Janus kinase inhibitors, are currently used for rheumatoid arthritis treatment. Therefore, any mediator identified through a greater understanding of the mechanisms involved in OA pathogenesis may be a potential target for OA treatment. Although there are an enormous number of mediators involved in the pathogenesis of OA, studies on the inhibition of these mediators using SMIs are limited. Theoretically, any SMI designed on the basis of information on the mediators in OA pathogenesis obtained through gain- and loss-of-function studies might have value. Thus, targeted therapies using further SMIs should be optimized for OA treatment in the future, which is an urgent and challenging task. In conclusion, SMIs have therapeutic potential in OA treatment. OA pathogenesis should be further explored as a potential target for OA treatment.
